# A mixture model with a reference-based automatic selection of components for disease classification from protein and/or gene expression levels

**DOI:** 10.1186/1471-2105-12-496

**Published:** 2011-12-30

**Authors:** Ivica Kopriva, Marko Filipović

**Affiliations:** 1 Division of Laser and Atomic R&D, Ruđer Bošković Institute, Bijenička cesta 54, 10000 Zagreb, Croatia

## Abstract

**Background:**

Bioinformatics data analysis is often using linear mixture model representing samples as additive mixture of components. Properly constrained blind matrix factorization methods extract those components using mixture samples only. However, automatic selection of extracted components to be retained for classification analysis remains an open issue.

**Results:**

The method proposed here is applied to well-studied protein and genomic datasets of ovarian, prostate and colon cancers to extract components for disease prediction. It achieves average sensitivities of: 96.2 (sd = 2.7%), 97.6% (sd = 2.8%) and 90.8% (sd = 5.5%) and average specificities of: 93.6% (sd = 4.1%), 99% (sd = 2.2%) and 79.4% (sd = 9.8%) in 100 independent two-fold cross-validations.

**Conclusions:**

We propose an additive mixture model of a sample for feature extraction using, in principle, sparseness constrained factorization on a sample-by-sample basis. As opposed to that, existing methods factorize complete dataset simultaneously. The sample model is composed of a reference sample representing control and/or case (disease) groups and a test sample. Each sample is decomposed into two or more components that are selected automatically (without using label information) as control specific, case specific and not differentially expressed (neutral). The number of components is determined by cross-validation. Automatic assignment of features (*m*/*z *ratios or genes) to particular component is based on thresholds estimated from each sample directly. Due to the locality of decomposition, the strength of the expression of each feature across the samples can vary. Yet, they will still be allocated to the related disease and/or control specific component. Since label information is not used in the selection process, case and control specific components can be used for classification. That is not the case with standard factorization methods. Moreover, the component selected by proposed method as disease specific can be interpreted as a *sub-mode *and retained for further analysis to identify potential biomarkers. As opposed to standard matrix factorization methods this can be achieved on a sample (experiment)-by-sample basis. Postulating one or more components with indifferent features enables their removal from disease and control specific components on a sample-by-sample basis. This yields selected components with reduced complexity and generally, it increases prediction accuracy.

## Background

**Figure 1:**
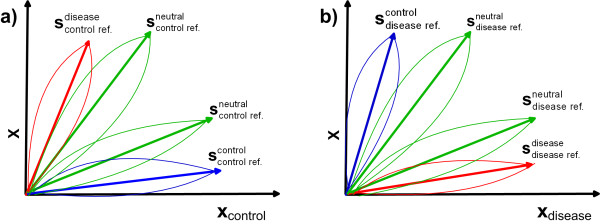
**Geometrical interpretation of the linear mixture model**. Concentration vectors of the linear mixture model comprised of control reference sample and test sample, (2a) and Figure 1a, i.e. disease reference sample and test sample, (2b) and Figure 1b, are confined in a first quadrant of the plane spanned by two mixture samples. Features (*m*/*z *ratios or genes) with prevailing concentration in disease sample are linearly combined into component associated with the red colour relative concentration vector. Likewise, features with prevailing concentration in control sample are combined linearly into component associated with the blue colour relative concentration vector. Features that are not differentially expressed are combined linearly into one or more neutral components associated with the green colour relative concentration vectors.

Bioinformatics data analysis is often based on the use of a linear mixture model (LMM) of a sample [1-15], whereas mixture is composed of components generated by unknown number of interfering sources. As an example, components can be generated during disease progression that causes cancerous cells to produce proteins and/or other molecules that can serve as early indicators (biomarkers) representing disease correlated chemical entities. Their correct identification may be very beneficial for an early detection and diagnosis of disease [16]. However, an identification of individual components within a sample is complicated by the fact that they can be "buried" within multiple substances. In addition to that, dynamic range of their concentrations can vary even several orders of magnitude [16], i.e., single components could no longer be recognizable [1]. Nevertheless, there are the algorithms able to extract either individual components or a group of components with similar concentrations within a sample. These algorithms are known under the name blind source separation (BSS) [17], and they commonly include independent component analysis (ICA) [18], and nonnegative matrix factorization (NMF) [19]. However, BSS methods perform unsupervised decomposition of the mixture samples. Thus, it is not clear which of the extracted components are to be retained for further prediction/classification analysis. To this end, several contributions toward solution of this problem have been published in [1-5,8]. In [1], a matrix factorization approach to the decomposition of infrared spectra of a sample is proposed taking into account class labels i.e., the classification phase and the components inference tasks are unified. Thus, the concept proposed in [1] is a classifier specific. It is formulated as the multiclass assignment problem where the number of components equals the number of classes and must be less than the number of samples available. As opposed to [1], the method proposed here selects automatically the case and control specific components on a sample-by-sample basis. Afterwards, these components can be used to train arbitrary classifier. In [2] gene expression profile is modelled as a linear superposition of three components comprised of up-regulated, down-regulated and differentially not expressed genes, whereas existence of two *fixed thresholds *is assumed to enable a decision to which of the three components the particular gene belongs. The thresholds are defined heuristically and in each specific case the optimal value must be obtained by cross-validation. Moreover, the upper threshold *c*_u _and the lower one *c*_l _are mutually related through *c*_u _= 1/*c*_l_. As opposed to that, the method proposed here decomposes each sample (experiment) into components comprised of up-regulated, down-regulated and not differentially expressed features using data adaptive thresholds. They are based on mixing angles of an innovative linear mixture model of a sample. The method proposed in [3] uses available sample labels (the clinical diagnosis of the experiments) to select component(s), extracted by independent component analysis (ICA) or nonnegative matrix factorization (NMF), for further analysis. ICA or NMF are used to factorize the whole dataset simultaneously and one selected component (gene expression mode for ICA and metagene for NMF) is used for further analysis related to gene marker extraction. This component cannot be used for classification. Alternatively, basis matrix with labelled column vectors (for ICA) or row vectors (for NMF) can be used for classification in which case the test sample needs to be projected to space spanned by the column/row vectors, respectively. However, in this case no feature extraction can be performed. As opposed to ICA/NMF method proposed in [3], the method proposed here extracts disease and control specific component from each sample separately. Since no label information is used in the selection process, extracted components can be used for classification and that is the goal in this paper. The disease specific component can, however, be also retained for further biomarker related analysis as in [3]. The important difference is that by the method proposed here such component can be obtained from each sample separately while the method in [3], as well as in [4,5,8], needs the whole dataset. The method [4] uses again ICA (the FastICA algorithm [20]) to factorize the microarray dataset. Extracted components (gene expression modes) were analyzed to discriminate between those with biological significance and those representing noise. However, biologically significant components can be used for further gene marker related analysis but not for classification. The reason is that, as in [3], the whole dataset composed of case and control samples is reduced to several biologically interesting components only. In the extreme case it can only be one such component. In [5] the JADE ICA algorithm is used to decompose whole dataset into components (gene expression modes). As in [3,4] these components cannot be used for classification. They are used for further decomposition into sub-modes to identify a regulating network in the problem considered there. We want to emphasize that the component selected as disease specific by the method proposed here can also be interpreted as a sub-mode and used for the similar type of analysis. However, since it is extracted from an individual and labelled sample it can be used for the classification as well. That is the main goal in this paper. The method in [8] again uses ICA (the maximum likelihood with natural gradient [18]) to extract components (gene expression modes). Similarly, as in [3-5] these components are not used for a classification. Instead, they are further analyzed by data clustering to determine biological relevance and extract gene markers. Similar types of comments as those discussed in relation to [3-5,8] can also be raised to other methods that use either ICA or NMF to extract components from the whole dataset, [6,7,10-12]. Hence, although related to the component selection methods [1,3-5,8] the method proposed here is dissimilar to all of them by the fact that it extracts most interesting components on a sample (experiment)-by-sample basis. To achieve this, the linear mixture model (LMM) used for components extraction is composed of a test sample and a reference sample representing control and/or case group. Hence, a test sample is, in principle, associated with two LMMs. Each LMM describes a sample as an additive mixture of two or more components. Two of them are selected automatically (no thresholds needed to be predefined) as case (disease) and control specific, while the rest are considered neutral i.e. not differentially expressed. Decomposition of each LMM is enabled by enforcing sparseness constraint on the components to be extracted. This implies that each feature (*m/z *ratio or gene) belongs to the two components at most (disease and neutral or, control and neutral). The model formally presumes that disease specific features are present in the prevailing concentration in disease samples as well as that control specific features are present in prevailing concentration in control samples. However, the features do not have to be expressed equally strong across the whole dataset in order to be selected as a part of disease or case specific components. It is this way due to the fact that decomposition is performed locally (on a sample-by-sample basis). This should prevent losing some important features for classification. Accordingly, the level of expression of indifferent features can also vary between the samples. Thus, postulating one or more components with indifferent features enables their removal that is sample adaptive. As opposed to that, existing methods try to optimize a single threshold for a whole dataset. Geometric interpretation of the LMM based on a reference sample enables automatic selection of disease and control specific components (Figure 1 in section 1.2), without using label information. Hence, the selected components can be further used for disease prediction. By postulating existence of one or more components with differentially not expressed features the complexity of the selected components can be controlled (see discussion in section 1.7), whereas the overall number of components is selected by cross-validation. Although the feature selection is the main goal of the proposed method, component extracted from the sample as disease specific can also be interpreted as a sub-mode as in [3,4]. It can be used for further biomarker identification related analysis. We see the linearity of the model used to describe a sample as a potential limitation of a proposed method. Although linear models dominate in bioinformatics, it has been discussed in [8] that nonlinear models might be more accurate description of biological processes. Assumption of an availability of a reference sample might also be seen as a potential weakness. Yet, we have demonstrated that in the absence of expert information the reference sample can be obtained by a simple average of all the samples within the same class. The proposed method is demonstrated in sections 1.4 to 1.7 on disease prediction problems using a computational model as well as on the experimental datasets related to a prediction of ovarian, prostate and colon cancers from protein and gene expression profiles.

## Methods

This section derives sparse component analysis (SCA) approach to unsupervised decomposition of protein (mass spectra) and gene expression profiles using a novel mixture model of a sample. The model enables automatic selection of the two of the extracted components as case and control specific. They are retained for classification. In what follows, the problem motivation and definition are presented first. Then, LMM of a sample is introduced and its interpretation is described. Afterwards, a two-stage implementation of the SCA algorithm is described and discussed in details.

### 1.1 Problem formulation

As mentioned previously, bioinformatics problems often deal with data containing components that are imprinted in a sample by several interfering sources. As an example, brief description of endocrine signalling system, secreting hormones into a blood stream, is given in [1]. Likewise, reference [21] describes how different organs imprint their substances (metabolites) into a urine sample. As pointed out in [1] and [16] disease samples are combinations of several co-regulated components (signals) originating from different sources (organs) and disease specific component is actually "buried" within a sample. Hence we are dealing with the two problems simultaneously: a sample decomposition (component inference) problem and a classification (disease prediction) problem that is based on sample decomposition. Thus, automatic selection of one or more extracted components is of practical importance. It is also important that component selection is done without a use of label information in which case it can be used for classification.

Matrix factorization is conveniently used in signal processing to solve decomposition problems [17-19]. It is assumed that data matrix **X **∈ ℝ^*N *× *K *^is comprised of *N *row vectors representing mixture samples, whereas each sample is further comprised of *K *features (*m*/*z *ratios or genes). It is also assumed that *N *samples are labelled: ${\left\{x_{n}\in {\mathbb{R}}^{k},y_{n}\in \left\{1,-1\right\}\right\}}_{n=1}^{N}$, where 1 denotes positive (disease) sample and -1 stands for a negative (control) sample. Data matrix **X **is modelled as a product of two factor matrices:

(1) X = AS

where **A **∈ ℝ^*N *× *M *^and **S **∈ ℝ^*M *× *K *^, and *M *represents an *unknown *number of components present in a sample. Each component ${\left\{s_{m}\in {\mathbb{R}}^{K}\right\}}_{m=1}^{M}$ is represented by a row vector of matrix **S**. Nonnegative relative concentration profiles ${\left\{a_{m}\in {\mathbb{R}}_{+}^{N}\right\}}_{m=1}^{M}$ are represented by column vectors of matrix **A **and are associated with the particular components. Here, it will be presented how innovative version of the LMM (1) of a sample ${\left\{x_{n}\in {\mathbb{R}}^{k}\right\}}_{m=1}^{M}$ enables automatic selection of the case (disease) and control specific components out of ${\left\{s_{m}\right\}}_{m=1}^{M}$ components extracted by unsupervised factorization method: a two stage SCA. The method will then be demonstrated on a computational model as well as on a cancer prediction problem using well known proteomic and genomic datasets.

### 1.2 Novel additive linear mixture model of a sample

The LMM (1) is widely used in various bioinformatics problems [1-15]. Unless constraints are imposed on **A **and/or **S**, the matrix factorization implied by (1) is not unique. Typical constraints involve non-Gaussianity and statistical independence between components by ICA algorithms [6,18], and non-negativity and sparseness constraints by NMF algorithms, [7,11,12,19,22,23]. In addition to that, many ICA algorithms, as well as many NMF algorithms, also require the *unknown *number of components *M *to be less than or equal to the number of mixture samples *N*.

Depending on the context, this constraint can be considered as restrictive. There are, however, ICA methods developed for the solution of underdetermined problems that are known as overcomplete ICA, see Chapter 16 in [18], as well as [24,25]. However, as discussed in details in [18], overcomplete ICA methods also assume that unknown components are sparse. The two exemplary overcomplete ICA methods based on sparseness assumption are described in [24] and [25]. In [24] it is assumed that components are sparse and approximately uncorrelated ("quasi-uncorrelated"). This basically means that each feature belongs to one component only. That is even a fairly stronger assumption than what is used by the method proposed here. Likewise, in maximum likelihood (ML) approach to the overcomplete problem in [25] it is assumed that marginal distributions of the components are Laplacian. In this case the component estimation problem (assuming the mixing matrix is estimated by clustering) is reduced to linear program with equality constraint. In other words, a probabilistic ML problem is converted into a deterministic linear programming task. Hence, the overcomplete ICA effectively becomes SCA. This further justifies our choice of the state-of-the-art SCA method (described in section 1.3), to be used in a component extraction task. Here, we propose a novel type of the LMM model which is composed of two samples only:

(2a) $$\left[\begin{array}{c} x_{\text{control}}\\ x \end{array}\right]=A_{\text{control}}S_{\text{control}}$$

(2b) $$\left[\begin{array}{c} x_{\text{disease}}\\ x \end{array}\right]=A_{\text{disease}}S_{\text{disease}}$$

The first sample is a reference sample representing control group, **x**_control _∈ ℝ*^K^*, in (2a) and case (disease) group, **x**_disease _∈ ℝ*^K^*, in (2b). The second sample is actual test sample: $x\in {\left\{x_{n}\in {\mathbb{R}}^{k}\right\}}_{n=1}^{N}$. Coefficients of matrices $A_{\text{control}}\in {\mathbb{R}}_{+}^{2\times M}$ and $A_{\text{disease}}\in {\mathbb{R}}_{+}^{2\times M}$ in (2a) and (2b) refer to the amount of relative concentration at which related components are present in the mixture samples **x **and **x**_control _in (2a) or **x **and **x**_disease _in (2b). Source matrices **S**_control _∈ ℝ^*M *× *K *^and **S**_disease _∈ ℝ^*M *× *K *^contain (as row vectors), disease- and control specific components and, possibly, differentially not expressed components. Number of components *M *is assumed to be greater than or equal to 2. Evidently, for *M *= 2 existence of differentially not expressed components is not postulated. Importance of postulating components with indifferent features is to obtain less complex disease and control specific components used for classification (see also discussion in section 1.7). These components absorb features that do not vary substantially across the sample population. These features are removed automatically from each sample. The concentration is relative due to the fact that BSS methods enable estimation of the mixing and source matrices up to the scaling constant only. Therefore, it is customary to constrain the column vectors of the mixing matrix to unit ℓ_2 _or ℓ_1 _norm. The LMM proposed here is built upon an implicit assumption that disease specific features (*m*/*z *ratios or genes) are present in prevailing concentration in disease specific samples and in minor concentration in control specific samples. As opposed to that, control specific features are present in prevailing concentration in control specific samples and in minor concentration in disease specific samples. Features that are not differentially expressed are present in similar concentrations in both control and disease specific samples. These groups of features constitute components, whereas similarity of their concentration profiles enables automatic selection of the components extracted by unsupervised factorization. The assumption on the prevailing concentrations of up- and down-regulated features needs to be understood in the relative sense. It is justified on the basis of locality of proposed method since the components are extracted on a sample-by-sample basis. Thus, to be allocated in the same component (a case or a control specific one) feature does not need to be expressed in each sample equally strong. Since the LMMs (2a)/(2b) considered here are comprised of two samples only the non-negative mixing vectors are confined in the first quadrant of the plane spanned by control reference sample and test sample, see Figure 1a, or by disease reference sample and test sample, see Figure 1b. Thus, upon decomposition of the LMM (2a) into *M *components, the one associated with the mixing vector that confines the maximal angle with respect to the axis defined by control reference sample is selected as a disease specific component, Figure 1a. As opposed to that, the one associated with the mixing vector that confines the minimal angle with respect to the axis defined by control reference sample is selected as a control specific component. When decomposition is performed with respect to a disease reference sample, LMM (2b), the logic for an angle-based automatic selection of disease and control specific components is the opposite, see Figure 1b. The components not selected as disease or control specific are considered neutral i.e. not differentially expressed. Thus, LMMs (2a)/(2b) enable automatic selection of the components extracted by unsupervised factorization of mixture samples. Unlike selection method presented in [2] that is based on fixed thresholds which need to be determined by cross-validation, the thresholds (mixing angles) used in the method presented here are sample adaptive. An assumption that each feature is contained in disease specific and one of the neutral components, or control specific and one of the neutral components, represents a sparseness constraint. It enables solution of the related BSS problems through, in principle, two-stage SCA method described in section 1.3. However, sparseness constraint is not justified by mathematical reasons only but also, as emphasized in [3,6,11,12], by the biological reasons. As noted in [6] this is necessary if underlying component (source signal) is going to be indicative of ongoing biological processes in a sample (cell, tissue, serum, etc.). The same conjecture has actually also been used in a three components based gene discovery method in [2]. In this respect, the sparseness constrained NMF methods for microarray data analysis proposed in [7,11,12] also assume the same working hypothesis. As discussed in [11,12], it is the sparseness constraint that enabled biological relevance of obtained results. In microarray data analysis enforcement of sparseness constraint is biologically justified due to the fact that more sparse **S **gives rise to metagenes (if factorization is performed by NMF), or to the expression modes (if factorization is performed by ICA), that comprise few dominantly co-expressed genes which may indicate good local features for specific disease [11]. A subtle interpretation of the reference-based mixture model (2a)/(2b) reveals its several profound characteristics. Since placement of the features to each of the two or more postulated components is based on sample adaptive thresholds (decomposition is localized), one gene (or *m*/*z *ratio) may be highly up-regulated in a case of one sample and significantly less expressed in a case of an another sample. Yet, if it is contained in prevailing concentration in both samples it will be contained in both cases in the component automatically selected as disease or control specific. Moreover, sample adaptive component (feature) selection enables that features selected as up- (or down)-regulated in one sample be less (or more) expressed than differentially not expressed features in another sample. Thus, extracted components selected as disease or control specific are composed of multiple features with different expression levels and joint discriminative power rather than of several (or even single) features only.

For disease prediction, disease and control specific components can be used to train a classifier. The reason is that in each LMM (2a)/(2b) they are extracted with respect to different reference samples and, thus, carry on different but specific information. Hence, proposed method yields four components to be retained for classifier training. In accordance with Figure 1 they are denoted as $s_{\text{control ref}\text{.;}n}^{\text{disease}}$, $s_{\text{control ref}\text{.;}n}^{\text{control}}$, $s_{\text{disease ref}\text{.;}n}^{\text{control}}$, and $s_{\text{disease ref}\text{.;}n}^{\text{disease}}$, where *n *denotes index of a test sample **x***_n _*used in current decomposition. Components extracted from *N *mixture samples, form four sets of labelled feature vectors as follows: ${\left\{s_{\text{control ref}\text{.;}n}^{\text{disease}},y_{n}\right\}}_{n=1}^{N}$, ${\left\{s_{\text{control ref}\text{.;}n}^{\text{control}},y_{n}\right\}}_{n=1}^{N}$, ${\left\{s_{\text{disease ref}\text{.;}n}^{\text{control}},y_{n}\right\}}_{n=1}^{N}$ and ${\left\{s_{\text{disease ref}\text{.;}n}^{\text{disease}},y_{n}\right\}}_{n=1}^{N}$. One or more classifiers can be trained on them and the one with the highest accuracy achieved through cross-validation is selected for a disease diagnosis.

Selection of the *unknown *number of components *M *is generally non-trivial problem in a matrix factorization and is the part of a model validation procedure. *M *is selected through cross-validation and postulated to be 2, 3, 4 or 5 because it directly determines the number of features used for classification. This follows from previously described interpretation of the LMM (2a) and (2b). Since disease prediction is based on four components selected as disease and control specific it is important that they are composed of features with the high discriminative power. It means that they should contain features which are truly disease or control specific. The component considered here as disease or control specific (as well as neutral) can actually be composed of features (*m/z *ratios or genes) belonging to multiple substances (metabolites, analytes) that share similar relative concentrations. This is practically important since it makes decomposition much less sensitive to an underestimation of the true total number of substances present in a sample. By setting the number of substances to predefined value *M*, proposed method is enforcing substances with similar concentrations to be linearly combined into one more complex components composed of disease, neutral or control specific features. Provided that concentration variability of these features across the samples is small, it would suffice to select overall number of components as *M *= 3 or even *M *= 2. (In the latter case, the existence of differentially not expressed features is not postulated at all). However, since we are dealing with biological samples it is more realistic to expect that relative concentrations could vary across the sample population. This is illustrated in Figures 1a and 1b by ellipsoids around vectors that represent *average *concentration profiles of each group of features (components). As seen from Figure 1, some features considered neutral can be present in the prevailing concentration in a certain number of samples than the features considered in a majority of the samples as disease (or control) specific. To partially remove such features from disease and/or control specific components, an unknown number of components *M *should be increased to *M *= 4 or perhaps even to *M *= 5. Thus, existence of two or three neutral components should be postulated. This is expected to yield less complex disease and control specific components and that is in agreement with the principle of parsimony (see also discussion in section 1.7). Model validation presented in section 1.4 suggests that this, indeed, is the case when concentration variability across the samples is significant. When it comes to the real world datasets, the information about number of components will not be known in advance. The strategy to comply with this uncertainty is to use the cross-validation and to verify whether increased number of components *M *indeed contributed to increased accuracy in disease prediction.

### 1.3 Sparse component analysis algorithm

Proposed feature extraction/component selection method is based on a decomposition of LMMs (2a)/(2b) comprised of two samples (reference sample and test sample) into *M *≥ 2 components. From the BSS point of view this yields determined BSS problem when *M *= 2 and underdetermined BSS problem, when *M *≥ 3 [26, 27, Chapter 10 in 17]. The enabling constraint for solving underdetermined BSS problems is a sparseness of the components and the methods are known under the common name as sparse component analysis (SCA) [26-29, Chapter 10 in 17]. As commented at the beginning of section 1.2 the overcomplete ICA, [Chapter 16 in 18, 24, 25], is basically reduced to SCA and also demands sparse sources. SCA has already been applied to microarray data analysis in [3,6,7,11,12]. It has also been used in [22,23] to extract more than two components from the two mixture samples of nuclear magnetic resonance and mass spectra. A sparseness constraint implies that each particular feature point *k *= 1, ...,*K *(*m/z *ratio or gene) belongs to the several components only. To this end, for the two-samples based LMMs (2a)/(2b) used here, it is assumed that each feature point belongs to at most two components: either disease specific and neutral or control specific and neutral. From the viewpoint of biology, a plausibility of this assumption has been elaborated before.

Algorithmic approaches used to solve underdetermined BSS problem associated with (2a)/(2b) belong to the two main categories: (*i*) estimating concentration/mixing matrix and component matrix simultaneously by minimizing data fidelity terms ${∥X-A_{\text{control}}S_{\text{control}}∥}_{F}^{2}$ or ${∥X-A_{\text{disease}}S_{\text{disease}}∥}_{F}^{2}$, where **X **follows from the left side of (2a) or (2b). A minimization is usually done through the alternating least square (ALS) methodology with sparseness constraint imposed on source matrices **S**_control _and **S**_disease_, [19,22,23,30-32]; (*ii*) estimating concentration/mixing matrices first by clustering and source/component matrices afterwards by solving underdetermined system of linear equations through minimization of the ℓ*_p _*norm, 0 <*p ≤ *1, of the column vectors **s***_k _*∈ ℝ*^M ^*of **S**_control _and **S**_disease_, [25-29,33-35]. As discussed in [6], a sparseness constrained minimization of the data fidelity term is sensitive to the choice of a sparseness constraint. On the other side, it has been recognized in [33-35] that accurate estimation of the concentration matrix enables accurate solution of even determined BSS problems. To this end, selection of feature points where only single component is present is of a special importance. At these points, feature vector and appropriate mixing vector are collinear. For example, if feature *k *belongs to component *m *then: **x***_k _*≈ **a***_m _s_mk_*. Thus, clustering of a set of single component points (SCPs) ought to yield an accurate estimate of the mixing matrix. Its columns are represented by cluster centroids. It has been demonstrated in [33] that such estimation of the mixing matrix, where hierarchical clustering was used, yields more accurate solution of determined BSS problem: **S **= *pinv*(**A**)**X**, than the one obtained by ICA algorithms. Thus, selection of SCPs is of an essential importance for accurate estimation of the mixing matrix. Such feature points are identified from the overall number of *K *points using geometric criterion based on the notion that at them real and imaginary parts of the mixture samples point either in the same or in the opposite direction [33,34]. Since protein (mass spectra) and gene expression levels are real sequences an analytic continuation [22] of mixture samples:

$x_{n}\mapsto {\tilde{x}}_{n}=x_{n}+\sqrt{-1}H\left(x_{n}\right)$ is used to obtain complex representation, where *H*(**x***_n_*) denotes Hilbert transform of **x***_n_*. The feature point *k *will be selected to the set of *J *SCPs provided that the following criterion is satisfied:

$$∥\frac{R{\left({\tilde{x}}_{k}\right)}^{\text{T}}I\left({\tilde{x}}_{k}\right)}{∥R\left({\tilde{x}}_{k}\right)∥∥I\left({\tilde{x}}_{k}\right)∥}∥\geq \cos\left(\Delta \theta \right)\;k\in \left\{1,...,K\right\}$$

where $R\left({\tilde{x}}_{k}\right)$ and $I\left({\tilde{x}}_{k}\right)$ denote real and imaginary part of ${\tilde{x}}_{k}$ respectively, 'T' denotes transpose operation, $∥R\left({\tilde{x}}_{k}\right)∥$ and $∥I\left({\tilde{x}}_{k}\right)∥$ denote ℓ_2_-norms of $R\left({\tilde{x}}_{k}\right)$ and $I\left({\tilde{x}}_{k}\right)$ while Δ*θ *stands for the angular displacement from direction of either 0 or π radians. Evidently, Δθ determines quality of the selected SCPs and, thus, accuracy of the estimation of the mixing matrices **A**_control _and **A**_disease_. Setting Δθ to a small value (e.g., to an equivalent of 1^0 ^) enforces, with an overwhelming probability, the selection of feature points that contain one component only. If, however, all the components are not present in at least one feature point alone it may occur that corresponding columns of the mixing matrices will be estimated inaccurately. This problem can be alleviated by increasing the value of Δθ in which case the selected feature points may not contain one component only, but may rather be composed of one dominant component and one or more components present in a small amount.

Thus, in practice, Δθ needs to be selected through a cross-validation. In the experiments described in sections 1.4 to 1.7, Δθ has been selected from the set of radians equivalent to {1^0^, 3^0^, 5^0^} together with a postulated number of components *M *and with a regularization parameter related to sparseness constraint imposed on **S**_control _and **S**_disease _(see eq. (3) below). Hierarchical clustering implemented by MATLAB clusterdata command (with a '*cosine*' distance metric and '*complete*' linkage option) has been used to cluster the set of selected *J *feature points with a single component belonging. Number of clusters has been set in advance to equal the postulated number of components *M*. Cluster centres represent estimated concentrations vectors ${\left\{a_{m}\in {\mathbb{R}}_{+}^{2}\right\}}_{m=1}^{M}$. It is also possible to use other clustering methods, such as *k*-means, as an alternative to hierarchical clustering. The problem with *k*-means, however, is that it is non-convex and its performance strongly depends on the initial value selected for cluster centroids. On the other side, hierarchical clustering produces repeatable result i.e. for a given set of SCPs it yields the same result for the mixing matrix in each run. Since the number of selected SCPs is modest, the computational complexity of hierarchical clustering approach is not too high. That is why hierarchical clustering is used to estimate the mixing matrices in (2a) and (2b). After mixing matrices are estimated, estimation of the component matrices proceeds by minimizing sparseness constrained cost functions:

(3a) $${\hat{S}}_{\text{control}}=\underset{\text{S}}{\min}\left\{\frac{1}{2}{∥{\hat{A}}_{\text{control}}S-\left[\begin{array}{c} x_{\text{control}}\\ x \end{array}\right]∥}_{F}^{2}+\lambda {∥S∥}_{1}\right\}$$

(3b) $${\hat{S}}_{\text{disease}}=\underset{\text{S}}{\min}\left\{\frac{1}{2}{∥{\hat{A}}_{\text{disease}}S-\left[\begin{array}{c} x_{\text{disease}}\\ x \end{array}\right]∥}_{F}^{2}+\lambda {∥S∥}_{1}\right\}$$

where the hat sign denotes estimates of the model variables **A**_control_/**A**_disease_ and **S**_control_/**S**_disease_. Problems (3) relate to the sparseness constrained solution of the underdetermined systems of linear equations. For a decomposition of gene expression profiles, a non-negativity constraint is additionally imposed on **S**: **S **≥ **0**. Problem (3) can be solved by the LASSO algorithm [36] or, by some other solver for underdetermined system of linear equations [37]. Here, for problem (3) we have used the iterative shrinkage thresholding (IST) type of method [38], with a MATLAB code available at [39]. This approach has been shown to be fast and it can be easily implemented in batch mode such as (3a)/(3b) i.e. as a solving of all *K *systems of equations simultaneously. In relation to standard IST methods, the method [38] has guaranteed better global rate of convergence. In addition to that, through the effect of iterations, it shrinks to zero small nonzero elements of **S **that are influenced by noise. This prevents them to determine level of sparseness of **S**. As discussed in [6] this shrinking operation is important in preventing selection of less sparse **S **over the sparse version of **S**. With non-negativity constraint **S **≥ **0 **problem (3) becomes a quadratic program. Thus, we have used a gradient descent with projection onto non-negative orthant: max(**0**,**S**). A sparsity of the solution is controlled by the parameter λ. There is a maximal value of λ (denoted by λ_max _here) above which the solution of the problems (3) is maximally sparse, i.e. it is equal to zero. Thus, in the experiments reported in sections 1.5 to 1.7 the value λ has been selected by cross-validation (together with Δθ and *M*) with respect to λ_max _as: λ∈{10^-2^·λ_max_, 10^-4^·λ_max_, 10-^6^·λ_max_}. We conclude this section by an observation that the situation suggested in [6]: **X **= **AS **= **A***^pseu^***S***^pseu^*, where (**A***^pseu^*, **S***^pseu^*) represents alternative factorization of **X **such that **S***^pseu ^*would be less sparse than **S**, during minimization of (3) cannot occur. That is due to IST algorithm [38] as well as due to accurate estimation of the mixing matrices that is enabled by clustering set of the SCPs. First, this is a consequence of the fact that a shrinking operation used by IST algorithm [38] imposes sparseness constraint of the type given by eq.(7) in [6]:

$$0\leq \sigma_{\tau }\left(s_{k}\right)=\frac{\text{number of elements of }s_{k}\leq \tau \cdot s_{k}^{\max}}{\text{number of elements of }s_{k}}\leq 1,\tau \in \left[0,1\right],$$

i.e. small nonzero elements of **s***_k _*are set to zero. This prevents selection of less sparse **S***^pseu ^*over sparser **S**. Second, SCA method used here is a two-stage method where **A **is estimated accurately by clustering on a set of SCPs. This, in addition to a sparseness measure discussed above, prevents estimate of **S **to deviate from the true value significantly. It is this way because when **S **is being estimated by means of IST algorithm the very estimate of **A **is fixed. As opposed to the case when **A **and **S **are estimated simultaneously, as in [6], an estimate of **A **can't now be adjusted by the algorithm to some value **A***^pseu ^*that will counteract changes in **S**. Hence, selecting **S***^pseu ^*would increase a data fidelity term in the cost function. Thus, situation as suggested in [6]: **X **= **AS **= **A***^pseu^***S***^pseu ^*can't occur. A proposed two-stage SCA approach to feature extraction/component selection is in a concise form presented in Table 1. A MATLAB code is posted in the Additional Material Files section accompanied with the paper as Additional File 1.

**Table 1:** A mixture model with a reference-based algorithm for feature extraction/component selection

**Figure 2:**
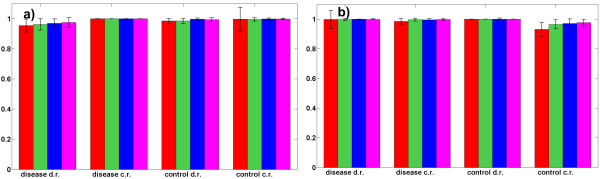
**model validation**. Sensitivities, Figure 2a, and specificities, Figure 2b, (with standard deviations as error bars) estimated by linear SVM classifier and 100 independent two-fold cross-validations using two disease specific and two control specific components. Components were extracted from the linear mixture models based on control reference (c.r.) sample, model (2a), and disease reference (d.r.) sample, model (2b), where each sample was comprised of ten orthogonal components containing *K=*15000 features. One component contained in prevailing concentration disease specific features, one control specific features and eight components contained features equally expressed in control and disease labelled samples. Relative concentration (expressed through a mixing angle) across the sample population has been: for disease specific features in the range of 50^0 ^to 89.99^0^; for differentially not expressed features in the range of 25^0 ^to 65^0^; and for control specific features in the range of 0.01^0 ^to 40^0^. Assumed overall number of components has been *M=*2 (red bars), *M *= 3 (green bars), *M=*4 (blue bars) and *M *= 5 (magenta bars).

**Inputs. ${\left\{x_{n}\in {\mathbb{R}}^{k},y_{n}\in \left\{1,-1\right\}\right\}}_{n=1}^{N}$**samples and sample labels, where *K *represents number of feature points (*m*/*z *ratios or genes).
**x**_control _∈ ℝ*^K ^*and **x**_disease _∈ ℝ*^K ^*representing control and disease (case) groups of samples.
**Nested two-fold cross-validation**. Parameters: single component points (SCPs) selection threshold in radian equivalents of Δ θ {1^0^, 3^0^, 5^0^}; regularization constant λ∈ {10^-2^λ_max_, 10^-4^λ_max_, 10^-6^λ_max_}; number of components *M *∈{2, 3, 4, 5}; parameters of selected classifier.
**Components selection from mixture samples**.
**1. $\forall x\in {\left\{x_{n}\in {\mathbb{R}}^{k}\right\}}_{n=1}^{N}$**form a linear mixture models (LMMs) (2a) and (2b).
**2**. For LMMs (2a)/(2b) select a set of single component points for a given Δθ.
**3**. On sets of SCPs use hierarchical clustering (other clustering methods can be used also) to estimate mixing matrices **A**_control _and **A**_disease _for a given *M*.
**4**. Estimate source matrices **S**_control _and **S**_disease _by solving (3a) and (3b) respectively for a given regularization parameter λ.
**5**. Use minimal and maximal mixing angles estimated from mixing matrices **A**control and **A**disease to select, following the logic illustrated in Fig. 2a and Fig. 2b, disease and control specific components: $s_{\text{control ref}\text{.;}n}^{\text{disease}}$, $s_{\text{control ref}\text{.;}n}^{\text{control}}$, $s_{\text{disease ref}\text{.;}n}^{\text{control}}$ and $s_{\text{disease ref}\text{.;}n}^{\text{disease}}$.
**End of component selection**.
**End of nested two-fold cross-validation**.

## Results and Discussion

This section presents model validation procedure. It is demonstrated how increased number of postulated components retains, or slightly improves, prediction accuracy when concentration variability of the features across the sample population is significant. Moreover, an increased number of postulated components yields the disease and control specific components used for classification with a smaller number of features. This is in an agreement with the principle of parsimony which states that less complex solution ought to be preferred over the more complex one. Proposed method for feature extraction/component selection is also applied to a prediction of ovarian, prostate and colon cancers from the three well-studied datasets. Prediction accuracy (sensitivity and specificity with standard deviations) is estimated by 100 independent two-fold cross-validations. Proposed SCA component selection method is compared (favourably) against state-of-the-art predictors tested on the same datasets including our implementation of methods proposed in [1,2]. Regarding our implementation of a predictive matrix factorization method [1], we have used the MATLAB fminsearch function to minimize the negative value of the target function suggested in [1] while selecting the threshold vector. We have set the TolFun to 10^-10^, the TolX to 10^-10 ^and the MaxFunEvals to 10,000. An initial value of the two-dimensional threshold vector has been set to [0 0]^T^. Regarding a gene discovery method proposed in [2] we have cross-validated three values of the threshold *c*_u _∈{2, 2.5, 3.0} (*c*_l _is set automatically *c*_l _= 1/*c*_u_). The best result is presented in section 1.7. Regarding a comparison of a proposed component selection method against many methods in sections 1.5 to 1.7, our intention has been to provide a brief description of the methods and to provide fair comparison given the fact that code for compared methods has not been available to us. That actually was the main reason for choosing a well known datasets such as in 1.5 to 1.7, since a rich list of published results exists for them. We are aware of the fact that results by many other methods were obtained by different cross-validation settings. Therefore, our reasoning is that fair comparison is possible as long as the results to be compared were obtained on the same datasets under conditions that favor less the method proposed here. That is the reason why we have chosen to perform two-fold cross-validation, since it is known to yield the least optimistic result. Thus, if such results are compared favorably against those obtained under milder (ten- and three-fold) cross-validation settings, conclusion can be made that proposed feature extraction/component selection method represents contribution to the field. As opposed to the two-fold cross-validation applied here, cross-validation details for many cited results were not specified. Sometimes ten-fold, or three-fold, cross-validations have been performed. Hence, it is believed that performance assessment of proposed component selection method is more realistic than performance of the majority of methods cited in comparative analysis. For each of the three types of cancers three classifiers were trained on four sets of extracted components: ${\left\{s_{\text{control ref}\text{.;}n}^{\text{disease}},y_{n}\right\}}_{n=1}^{N}$, ${\left\{s_{\text{control ref}\text{.;}n}^{\text{control}},y_{n}\right\}}_{n=1}^{N}$, ${\left\{s_{\text{disease ref}\text{.;}n}^{\text{control}},y_{n}\right\}}_{n=1}^{N}$ and ${\left\{s_{\text{disease ref}\text{.;}n}^{\text{disease}},y_{n}\right\}}_{n=1}^{N}$. The three classifiers used were linear SVM and nonlinear SVM with radial basis function (RBF) and polynomial kernels [40], with *C *= 1. Parameters of the nonlinear SVM classifiers were selected by cross-validation. Prior to the classification, the sets of extracted components were standardized to zero mean and unit variance. Although the standardization across the features is used more often, a standardization across the components (they coincide with the samples from which they were extracted) has been performed here. It yielded much better accuracy and such a fact has also been observed in Chapter 18 in [41], where in microarray data analysis standardization across the samples has also been preferred over standardization across the features. In comparative performance analysis presented in Tables 2, 3 and 4 the best result (obtained by a nested two-fold cross-validation with respect to parameters of the classifiers, single component selection threshold Δθ, regularization constant λ and postulated number of components *M *) on all four sets of selected components has been used to represent component selection method proposed here. Since many components extracted by other combinations of the parameters yielded also good prediction accuracy we have posted complete results in the Additional Material Files section (Additional Files 2, 3, 4 and 5) accompanied with the paper. Reference samples used to represent disease and control groups were obtained by averaging all the samples in disease group, $x_{\text{disease}}=\frac{1}{N_{1}}\sum_{i=1}^{N_{1}}x_{i}$ where $x_{i}\in {\left\{x_{n}:y_{n}=1\right\}}_{n=1}^{N_{1}}$, and control group, $x_{\text{control}}=\frac{1}{N_{2}}\sum_{i=1}^{N_{2}}x_{i}$ where $x_{i}\in {\left\{x_{n}:y_{n}=1\right\}}_{n=1}^{N_{2}}$ and *N*_1 _+ *N*_2 _= *N*. We thought this is the most fair approach in the absence of any *prior *information that could suggest which labelled sample could serve as a gold standard. We conclude this section by providing assessment of the computational complexity of proposed method. It has been implemented in MATLAB 7.7 environment on a desktop computer based on 3 GHz dual core processor and 2 GB of RAM. Processing of proteomic and genomic datasets used in sections 1.5 to 1.7 took 10, 7 and 3 minutes respectively.

**Table 2:** Comparative performance results in ovarian cancer prediction. Sensitivities and specificities were estimated by 100 two-fold cross-validations (standard deviations are in brackets).

Method	Sensitivity/Specificity/Accuracy
Proposed method *M *= 3, Δθ = 5^0^ λ = 10^-4^λ_max_ Linear SVM	Sensitivity: 96.2 (2.7)%; specificity: 93.6 (4.1)%; accuracy: 94.9% Control specific component extracted with respect to a cancer reference sample.

Proposed method *M *= 4, Δθ = 3^0^ λ = 10^-6^λ_max_ Linear SVM	Sensitivity: 95.4 (3)%; specificity: 94 (3.7)%; accuracy:94.7% Control specific component extracted with respect to a cancer reference sample.

[1]	Sensitivity: 81.4 (7.1)%; specificity: 71.7 (6.6)%

[42]	Sensitivity: 100%; specificity: 95% (one partition only: 50/50 training; 66/50 test).

[44]	Accuracy averaged over 10 ten-fold partitions: 98-99% (sd: 0.3-0.8)

[13]	Sensitivity: 98%, specificity: 95%, two-fold CV with 100 partitions.

[45]	Average error rate of 4.1% with three-fold CV.

**Table 3:** Comparative performance results in prostate cancer prediction. Sensitivities and specificities were estimated by 100 two-fold cross-validations (standard deviations are in brackets).

Methods	Sensitivity/Specificity/Accuracy
Proposed method *M *= 5, Δθ = 1^0^ λ = 10^-4^λ_max _Linear SVM	Sensitivity: 97.6 (2.8)%; specificity: 99 (2.2)%; accuracy: 98.3% Control specific component extracted with respect to a cancer reference sample.

Proposed method *M *= 4, Δθ = 1^0^ λ = 10^-4^λ_max _Linear SVM	Sensitivity: 97.7 (2.3)%; specificity: 98 (2.4)%; accuracy: 97.9% Control specific component extracted with respect to a cancer reference sample.

[1]	Sensitivity: 86 (6.6)%; specificity: 67.8(12.9)%; accuracy: 76.9%.

[46]	Sensitivity: 94.7%; specificity: 75.9%; accuracy: 85.3%. 253 benign and 69 cancers. Results were obtained on independent test set comprised of 38 cancers and 228 benign samples.

[47]	Sensitivity: 97.1%; specificity: 96.8%; accuracy: 97%. 253 benign and 69 cancers. Cross-validation details not reported.

[45]	Average error rate of 28.97 on four class problem with three-fold cross-validation.

**Table 4:** Comparative performance results in colon cancer prediction. Sensitivities and specificities were estimated by 100 two-fold cross-validations (standard deviations are in brackets).

Methods	Sensitivity/Specificity/Accuracy
Proposed method *M *= 2, Δθ = 1^0^ RBF SVM (σ^2 ^= 1200, C = 1)	Sensitivity: 90.8 (5.5)%, specificity: 79.4 (9.8)%; accuracy: 85.1% Control specific component extracted with respect to a cancer reference sample.

Proposed method *M *= 4, Δθ = 5^0 ^λ = 10^-2^λ_max_ RBF SVM (σ^2 ^= 1000, C = 1)	Sensitivity: 89.8 (6.2)%, specificity: 78.6 (12.8)%; accuracy: 84.2%. Control specific component extracted with respect to a control reference sample.

[1]	Sensitivity: 89.7 (6.4)%, specificity: 84.3 (8.4)%; accuracy = 87%. 100 two-fold cross-validations.

[2]	Sensitivity: 92.1 (4.7)%, specificity: 85 (10.1)%; accuracy: 88.55%. 100 two-fold cross-validations. *c*u = 2.0.

[48]	Sensitivity: 92-95% calculated from Figure 5. Specificity not reported.

[15]	Accuracy 85%. Cross-validation details not reported.

[50]	Accuracy 82.5%, ten-fold cross-validation (RFE with linear SVM).

[51]	Accuracy 88.84%, two-fold cross-validation (RFE with linear SVM and optimized penalty parameter C).

### 1.4 Model validation

This section presents model validation results obtained on simulated data using LMM (2a)/(2b). To this end, each mixture sample has been composed of ten orthogonal components comprised of *K *= 15000 features. The orthogonality implies that each feature belongs to one component only. By a convention, the first component has been selected to contain disease specific features, the tenth component to contain control specific features and the components two to nine contain features that are not differentially expressed and share similar concentrations in control and disease labelled samples. A concentration variability across the sample population is simulated using the following model for disease group of samples:

$$x_{n}=\sum_{m-1}^{M}\sin^{2}\left(\theta_{nm}\right)s_{m}$$

and for control group of samples:

(4) $$x_{n}=\sum_{m-1}^{M}\cos^{2}\left(\theta_{nm}\right)s_{m}$$

Thus, by controlling the mixing angles ${\left\{\theta_{nm}\right\}}_{n=1,m=1}^{N,M}$ the amount of a concentration of each component in disease and control samples is controlled. Also amount of concentration variability is controlled by selecting ${\left\{\theta_{nm}\right\}}_{n=1,m=1}^{N,M}$ to be confined within (non-) overlapping angular sectors. Note that (4) implies that component **s***_m _*is contained in a related disease and control samples in overall concentration of 100%. To simulate biological variability between the samples, the relative concentration has been varied across the sample population, where disease and control groups contained 100 samples each. The concentration vectors were overlapping in the mixing angle domain i.e. a concentration vector for disease specific features was confined in the sector of [50^0^, 89.99^0^], for the neutral features it was in the sector of [25^0^,65^0^] and for control specific features it was confined in the sector of [0.01^0^,40^0^]. Thus, amount of overlap between concentration profiles was significant, implying that in many cases neutral features were contained in greater concentrations in disease labelled samples than disease specific features, as well as that neutral features were contained in greater concentrations in control labelled samples than control specific features. Figures 2a and 2b show disease prediction results using four extracted disease and control specific components with the postulated overall number of components equal to *M *= 2 (red bars), *M *= 3 (green bars), *M *= 4 (blue bars) and *M *= 5 (magenta bars). Reference samples used in LMM (2a)/(2b) were obtained by averaging all the samples in control i.e. disease group. Results reported in terms of sensitivity (Figure 2a) and specificity (Figure 2b) were obtained by the linear support vector machine (SVM) classifier using 100 independent two-fold cross-validations. SCPs selection parameter has been set to Δθ = 3^0 ^and sparseness regularization parameter in (3a)/(3b) to λ = 10^-6^·λ_max_. These parameters were not selected through cross-validation since the purpose of the computational experiment has been to evaluate influence of the assumed number of components *M *to the prediction accuracy when concentration varies across the sample population. The presented results demonstrate that greater number of postulated components does not decrease prediction accuracy (in the average it is even slightly increased). However, increased number of postulated components *M *reduces the number of features contained in disease and control specific components selected for classification. As discussed previously, a greater *M *yields less complex disease and control specific components. Following the principle of parsimony such solution should be preferred over the more complex ones that are obtained for smaller *M*. Thus, selected disease and control specific components are expected to be more discriminative and less sensitive to over-fitting when the number of postulated components is increased. In practical implementation of the proposed approach to component selection the optimal number of overall components needs to be evaluated by a cross-validation. In the three real world experiments reported below the number of components has been selected by cross-validation from *M *∈ {2, 3, 4, 5}. If a prediction accuracy achieved for the two values of *M *is approximately equal, it is better to prefer components extracted from the samples with a greater value of *M*.

### 1.5 Ovarian cancer prediction from a protein mass spectra

**Figure 3:**
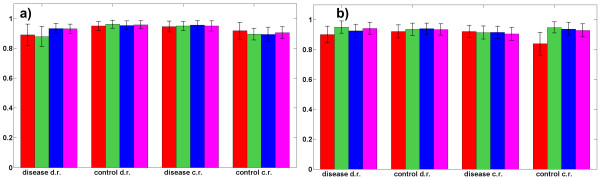
**ovarian cancer prediction**. Sensitivities (a) and specificities (b) (with standard deviations as error bars) estimated in ovarian cancer prediction from protein expression levels using 100 independent two-fold cross-validations and linear SVM classifier. Four sets of selected components were extracted by SCA-based factorization using LMMs (2a) and (2b) with control reference (c.r.) and disease reference (d.r.) samples respectively, where the overall number of components *M *has been set to 2 (red bars), 3 (green bars), 4 (blue bars) and 5 (magenta bars). Optimal values of the parameters λ and Δθ were used for each *M*. Performance improvement is visible when number of components is increased from 2 to 3, 4 or 5.

Low resolution surface-enhanced laser desorption ionization time-of-flight (SELDI-TOF) mass spectra of 100 controls and 100 cases have been used for ovarian cancer prediction study [42]. See also the website of the clinical proteomics program of the National Cancer Institute (NCI), [43], where the used dataset is labelled as "Ovarian 4-3-02". All spectra were baseline corrected. Thus, some intensities have negative values. Table 2 presents the best result obtained by the proposed SCA-based component selection method together with results obtained for the same dataset by competing methods reported in cited references as well as by predictive factorization method proposed in [1]. Described SCA method has been used to extract four sets of components with the overall number of components *M *assumed to be 2, 3, 4 and 5. Figure 3 shows sensitivities and specificities estimated by 100 independent two-fold cross-validations using linear SVM classifier which yielded the best results compared against nonlinear SVM classifiers based on polynomial and RBF kernels. Performance improvement is visible when assumed number of components is increased from 2 to 3, 4 or 5. The error bars are dictated by the sample size and would decrease with a larger sample. Thus, the mean values should be looked at to observe the trend in performance as a function of *M*. The best result (shown in Table 2) has been obtained with the linear SVM classifier for *M *= 3 with sensitivity of 96.2% and specificity of 93.6%, but results with the very similar quality have been obtained for several combinations of the parameters *M*, Δθ and λ, see Figure 3, most notably *M *= 4 (see second column in Table 2 and the Additional File 2). As seen in Table 2, only [13] reported better result for a two-fold cross-validation with the same number of partitions. There, a combination of genetic algorithm and *k*-nearest neighbours method, originally developed for mining of high-dimensional microarray gene expression data, has been used for analysis of proteomics data. However, the method [13] is tested on proteomic ovarian cancer dataset only, while the method proposed here exhibited excellent performance in prediction of prostate cancer from proteomic data (reported in section 1.6), as well as on colon cancer from genomic data (presented in section 1.7). The method shown in [42] used 50 samples from the control group and 50 samples from the ovarian cancer group to discover a pattern that discriminated cancer from non-cancer group. This pattern has then been used to classify an independent set of 50 samples with ovarian cancer and 66 samples unaffected by ovarian cancer. In [44], a fuzzy rule based classifier fusion is proposed for feature selection and classification (diagnosis) of protein mass spectra based ovarian cancer. Demonstrated accuracy of 98-99% has been estimated through 10 ten-fold cross-validations (as opposed to 100 two-fold cross-validations used here). Moreover, as demonstrated in sections 1.6 and 1.7, the method proposed here exhibited good performance on diagnosis of prostate and colon cancers from proteomic and gene expression levels, respectively. In [45], a clustering based method for feature selection from mass spectrometry data is derived by combining *k*-means clustering and genetic algorithm. The method exhibited an accuracy of 95.8% (error rate 4.1%), but this has been assessed through three-fold cross-validations (as opposed to two-fold cross-validations used here).

### 1.6 Prostate cancer prediction from a protein mass spectra

**Figure 4:**
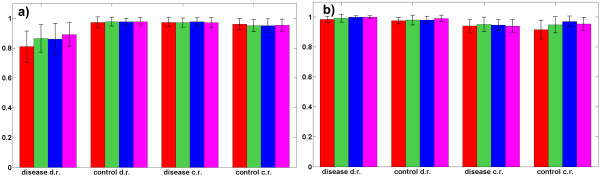
**prostate cancer prediction**. Sensitivities (a) and specificities (b) (with standard deviations as error bars) estimated in prostate cancer prediction from protein expression levels using 100 independent two-fold cross-validations and linear SVM classifier. Four sets of selected components were extracted by SCA-based factorization using LMMs (2a) and (2b) with control reference (c.r.) and disease reference (d.r.) samples respectively, where the overall number of components *M *has been set to 2 (red bars), 3 (green bars), 4 (blue bars) and 5 (magenta bars). Optimal values of the parameters λ and Δθwere used for each *M*. Performance improvement is visible when number of components is increased from 2 to 5.

Low resolution SELDI-TOF mass spectra of 63 controls: no evidence of cancer with prostate-specific antigen (PSA)<1, and 69 cases (prostate cancers): 26 with 4<PSA<10 and 43 with PSA>10, have been used for prostate cancer prediction study [46]. There are additional 190 control samples with benign cancer (4<PSA<10) available as well (see the website of the clinical proteomics program of the NCI, [43]), in dataset labelled as "JNCI_Data_7-3-02". However, in the two-class comparative performance analysis problem reported here these samples were not used. Proposed SCA-based method has been used to extract four sets of components with the overall number of components *M *assumed to be 2, 3, 4 and 5. The best result has been achieved for *M *= 5 with sensitivity of 97.6% and specificity of 99%, but results with the very similar quality have been obtained for several combinations of the parameters *M*, Δθ and λ, (see Figure 4 and the Additional File 3). Table 3 presents two best results achieved by the proposed SCA-based approach to component selection together with the results obtained by competing methods reported in cited references. Linear SVM classifier yielded the best results when compared against nonlinear SVM classifiers based on polynomial and RBF kernels. According to Table 3, comparable result (although slightly worse) is in the reference [47] only. The method [47] is proposed for analysis of mass spectra for screening of prostate cancer. The system is composed of three stages: a feature selection using statistical significance test, a classification by radial basis function and probabilistic neural networks and an optimization of the results through the receiver-operating-characteristic analysis. The method achieved sensitivity 97.1% and specificity 96.8% but the cross-validation setting has not been described in details. In [46], the training group has been used to discover a pattern that discriminated cancer from non-cancer group. This pattern has then been used to classify an independent set of 38 patients with the prostate cancer and 228 patients with the benign conditions. The obtained specificity is low. The predictive matrix factorization method [1] yielded significantly worse result than the method proposed here. In [45] a clustering based method for feature selection from mass spectrometry data is derived combining *k*-means clustering and genetic algorithm. Despite a three-fold cross-validation, the reported error was 28.97%. Figure 4 shows sensitivities and specificities estimated by 100 independent two-fold cross-validations using linear SVM classifier on components selected by the method proposed here. For each *M *the optimal values of the parameters λ and Δθ (obtained by cross-validation) have been used to obtain results shown in Figure 4. Increasing a postulated number of components from 2 to 5 increased accuracy from 97.4% to 98.3%. Thus, better accuracy is achieved with the smaller number of features (*m*/*z *ratios) contained in selected components.

### 1.7 Colon cancer prediction from gene expression profiles

**Figure 5:**
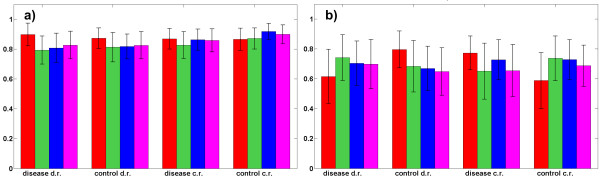
**colon cancer prediction**. Sensitivities (a) and specificities (b) (with standard deviations as error bars) estimated in colon cancer prediction from gene expression levels using 100 independent two-fold cross-validations and linear SVM classifier. Four sets of selected components were extracted by using LMMs (2a) and (2b) with control reference (c.r.) and disease reference (d.r.) samples respectively, where the overall number of components *M *has been set to 2 (red bars), 3 (green bars), 4 (blue bars) and 5 (magenta bars). Optimal values of the parameters λ and Δθwere used for each *M*. Increasing number of components *M *did not decrease prediction accuracy but did reduce the number of features (genes) in components used for classification (see Figure 6).

**Figure 6:**
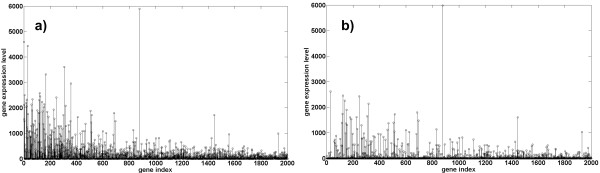
**colon cancer feature vectors**. Component containing up-regulated genes extracted from a cancerous sample w.r.t. to a control reference sample using LMM (2a): a) assumed number of components *M *= 2; b) assumed number of components *M *= 4.

Gene expression profiles of 40 colon cancer and 22 normal colon tissue samples obtained by an Affymetrix oligonucleotide array [48], have been also used for validation and comparative performance analysis of proposed feature extraction method. Gene expression profiles have been downloaded from [49]. Original data produced by oligonucleotide array contained more than 6500 genes but only 2000 high-intensity genes have been used for cluster analysis in [48] and are provided for download on the cited website. The proposed SCA-based approach to feature extraction/component selection has been used to extract four sets of components with up- and down-regulated genes and with the overall number of components *M *assumed to be 2, 3, 4 and 5. The linear SVM classifier has been applied to groups of the four sets of selected components extracted from gene expression levels for specific combinations of parameters Δθ, λ and *M*. The best result in terms of sensitivity and specificity for each *M *has been selected and shown in Figure 5. The complete list of results obtained by linear SVM classifier is presented in the Additional File 4. An increased number of postulated components *M *did not decrease accuracy but it yielded components selected for classification with reduced number of genes. This is verified in Figure 6 which shows component with up-regulated genes $s_{\text{control}}^{\text{disease}}$ extracted from a cancer labelled sample w.r.t. the control reference for assumed number of components *M *= 2 and *M *= 4. Thus, it is confirmed again that an increased *M *yields less complex components that (following the principle of parsimony), should be preferred over the more complex ones obtained by smaller *M*. In order to (possibly) increase the prediction accuracy, we have applied nonlinear, polynomial and RBF SVM classifiers to the two groups of the four sets of components that yielded the best results with the linear SVM classifier: *M *= 2 (Δθ = 1^0^) and *M *= 4 (λ = 10^-2^λmax and Δθ = 5^0^). The polynomial SVM classifier has been cross-validated for degree of the polynomial equal to d = 2, 3 and 4. The RBF SVM classifier $\kappa \left(x,y\right)=\exp\left(-{∥x-y∥}_{2}^{2}/2\sigma^{2}\right)$ has been cross-validated for the variance σ^2 ^in the range 5 × 10^2 ^to 1.5 × 10^3 ^in steps of 10^2^. The best result has been obtained with σ^2 ^= 1.2 × 10^3 ^for *M *= 2 and with σ^2 ^= 1.0 × 10^3 ^for *M *= 4. An achieved accuracy is comparable with the accuracy obtained by other state-of-the-art results reported. That is shown in Table 4 as well as in the Additional File 5. A predictive matrix factorization method [1] yielded slightly better results here, but it has shown significantly worse result in the cases of ovarian (see Table 2) and prostate (see Table 3) cancers. Gene discovery method [2] has been applied for three values of the threshold *c*_u _∈ {2, 2.5, 3} used to select up-regulated genes. Maximum *a posteriori *probability has been used for an assignment of genes to each of the three components containing up-, down regulated and differentially not expressed genes. Thus for each threshold value the two components were obtained for training a classifier. The logarithm with the base 10 has been applied to gene folding values prior gene discovery/selection took place. The best result reported in Table 4 has been obtained for a component containing up-regulated genes with *c*_u _= 2.0 and an RBF SVM classifier, whereas σ^2 ^has been cross-validated in the range 10^2 ^to 10^3 ^in steps of 10^2^. The best result has been obtained for σ^2 ^= 5 × 10^2^. The gene discovery method [2] outperformed slightly the method proposed here. However as opposed to the proposed method, the gene discovery method [2] is not applicable to the analysis of mass spectra. The gene selection method in [15] is a model driven trying to take into account the genes' group behaviours and interactions by developing an ensemble dependence model (EDM). The microarray dataset is clustered first. The EDM is based on modelling dependencies that represent inter-cluster relationships. Inter-cluster dependence matrix is the basis for discrimination between cancerous and non-cancerous samples. Classification accuracy of 85% reported in [15] is very close to the one obtained by the SCA-based method proposed here. However, while SCA-based performance has been assessed through two-fold cross-validation, no cross-validation details were reported in [15]. Similarly, sensitivity had to be estimated indirectly from Figure 5 in [48]. The method in [50] combines a recursive feature extraction and the linear SVM to yield accuracy of 82.5%. This is also less accurate than what has been achieved by the method proposed. Moreover, the very accuracy reported in [50] has been assessed by a ten-fold cross-validation only and that is known to yield a too optimistic performance assessment. In this regard accuracy reported in [51] can be taken closer to the realistic one since it has been assessed by two-fold cross-validation. This method, as [50], again combines recursive feature elimination with the SVM, but it is taking additionally into account the parameter *C*. A reported accuracy of 88.84% is slightly better than the one obtained by the method proposed here. However, the proposed method is a classifier independent one and, as demonstrated in sections 1.5 and 1.6, it yields good results on cancer diagnosis from proteomic datasets as well.

## Conclusions

This work presents a feature extraction/component selection method based on innovative additive linear mixture model of a sample (protein or gene expression levels represented respectively by mass spectra or microarray data) and sparseness constrained factorization that operates on a sample(experiment)-by-sample basis. That is different in respect to the existing methods which factorize complete dataset simultaneously. The sample model is comprised of a test sample and a reference sample representing disease and/or control group. Each sample is decomposed into several components selected automatically (the number is determined by cross-validation), without using label information, as disease-, control specific and differentially not expressed. An automatic selection is based on mixing angles which are estimated from each sample directly. Hence, due to the locality of decomposition, the strength of the expression of each feature can vary from sample to sample. However, the feature can still be allocated to the same (disease or control specific) component in different samples. As opposed to that, feature allocation/selection algorithms that operate on a whole dataset simultaneously try to optimize a single threshold for the whole dataset. Selected components can be used for classification due to the fact that labelled information is not used in the selection. Moreover, disease specific component(s) can also be used for further biomarker related analysis. As opposed to the existing matrix factorization methods, such disease specific component can be obtained from one sample (experiment) only. By postulating one or more components with differentially not expressed features the method yields less complex disease and control specific components that are composed of smaller number of features with higher discriminative power. This has been demonstrated to improve prediction accuracy. Moreover, decomposing sample with one or more components with indifferent features performs (indirectly) sample adaptive preprocessing related to removal of features that do not significantly vary across the sample population. The proposed feature extraction/component selection method is demonstrated on the real world proteomic datasets used for prediction of the ovarian and prostate cancers as well as on the genomic dataset used for the colon cancer prediction. Results obtained by 100 two-fold cross-validations are compared favourably against most of the state-of-the-art methods cited in the literature and used for cancer prediction on the same datasets.

## Authors' contributions

IK has proposed novel linear mixture model of the samples and methodology for automatic selection of disease and control specific components extracted from the samples by means of sparse component analysis. He also has been performed model validation and implemented the clustering phase of the sparse component analysis method. MF implemented iterative thresholding based shrinkage algorithm for extraction of the components and performed cross-validation based component extraction and classification. All authors read and approved the final manuscript.

## Supplementary Material

Additional file 1 **code with implementation of proposed feature extraction/component selection method**. Click here for file

Additional file 2 **classification results obtained by the linear SVM applied to disease and control specific components extracted from the ovarian cancer dataset for various combination of parameters *M*, λ and Δθ**. Click here for file

Additional file 3 **classification results obtained by the linear SVM applied to disease and control specific components extracted from the prostate cancer dataset for various combination of parameters *M*, λ and Δθ**. Click here for file

Additional file 4 **classification results obtained by the linear SVM applied to disease and control specific components extracted from the colon cancer dataset for various combination of parameters *M*, λ and Δθ**. Click here for file

Additional file 5 **best classification results obtained by the RBF SVM applied to disease and control specific components extracted from the colon cancer dataset for *M *= 4, λ = 10^-2^λ_max _and Δθ = 5^0 ^and *M *= 2 and Δθ = 1^0^**. Click here for file
